# Supplementary material for: Structural Aspects and Intermolecular Energy for Some Short Testosterone Esters
Source: Materials (Basel). 2022 Oct 17;15(20):7245. doi: 10.3390/ma15207245 (PMC9611952; doi:10.3390/ma15207245)
Supplement: Supplementary file 1 [file materials-15-07245-s001.zip › materials-1935035-supplementary.pdf]

Supporting information

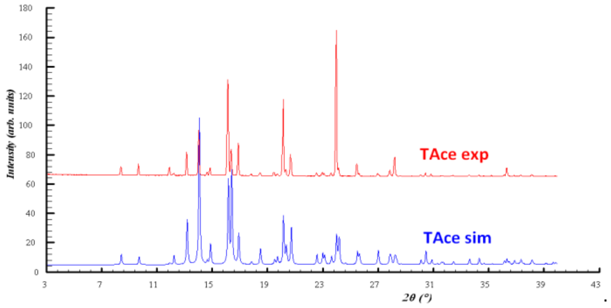

(a)

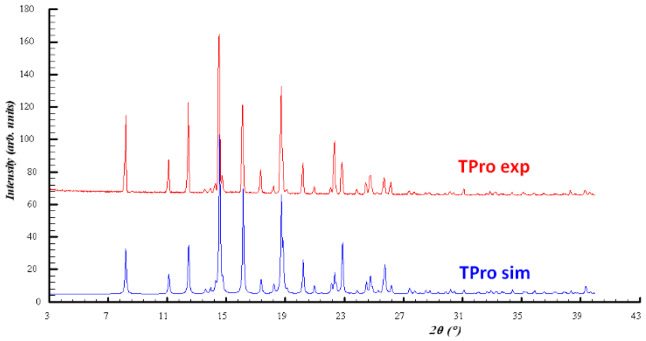

(b)

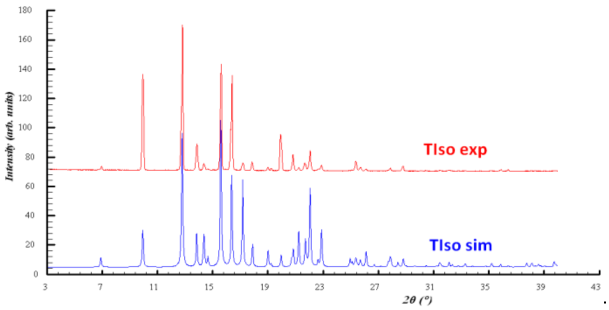

(c)

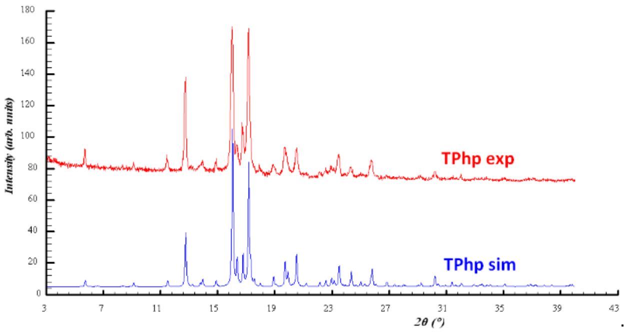

(d)

Figure S1. Experimental and simulated XRPD patterns comparison: TAce (a), TPro (b), TIso (c), TPhp (d)

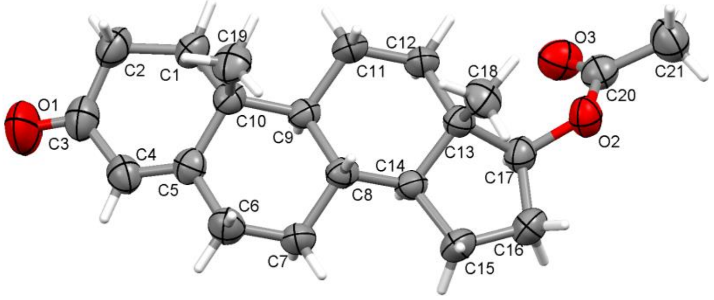

(a)

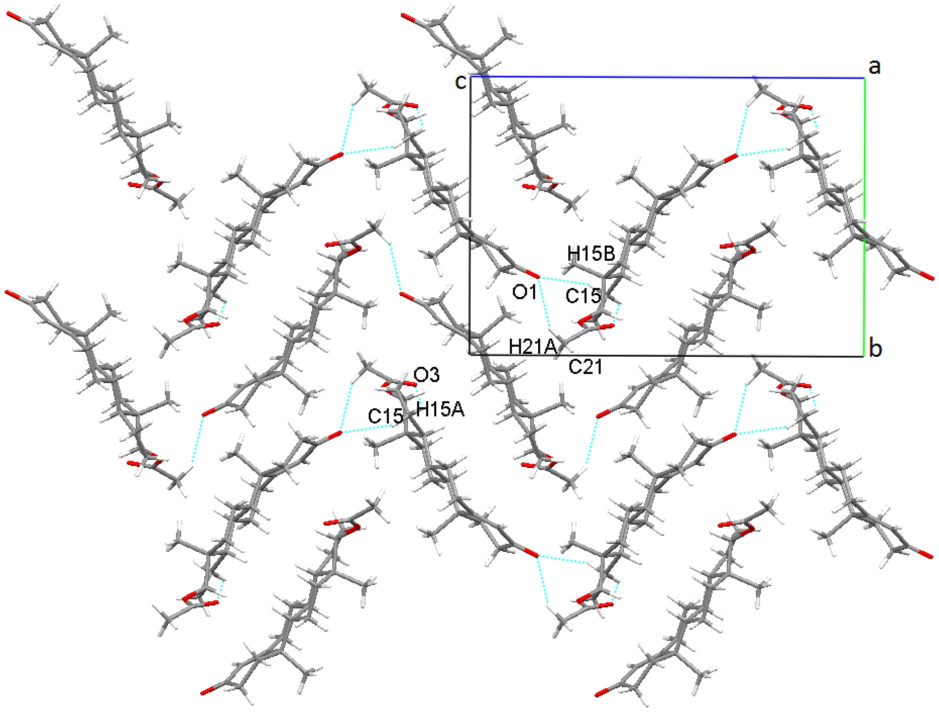

(b)

Figure S2. Asymmetric unit of TAce presenting non-hydrogen atoms at 50% probability level (a) Overall packing diagram along a-axis (b)

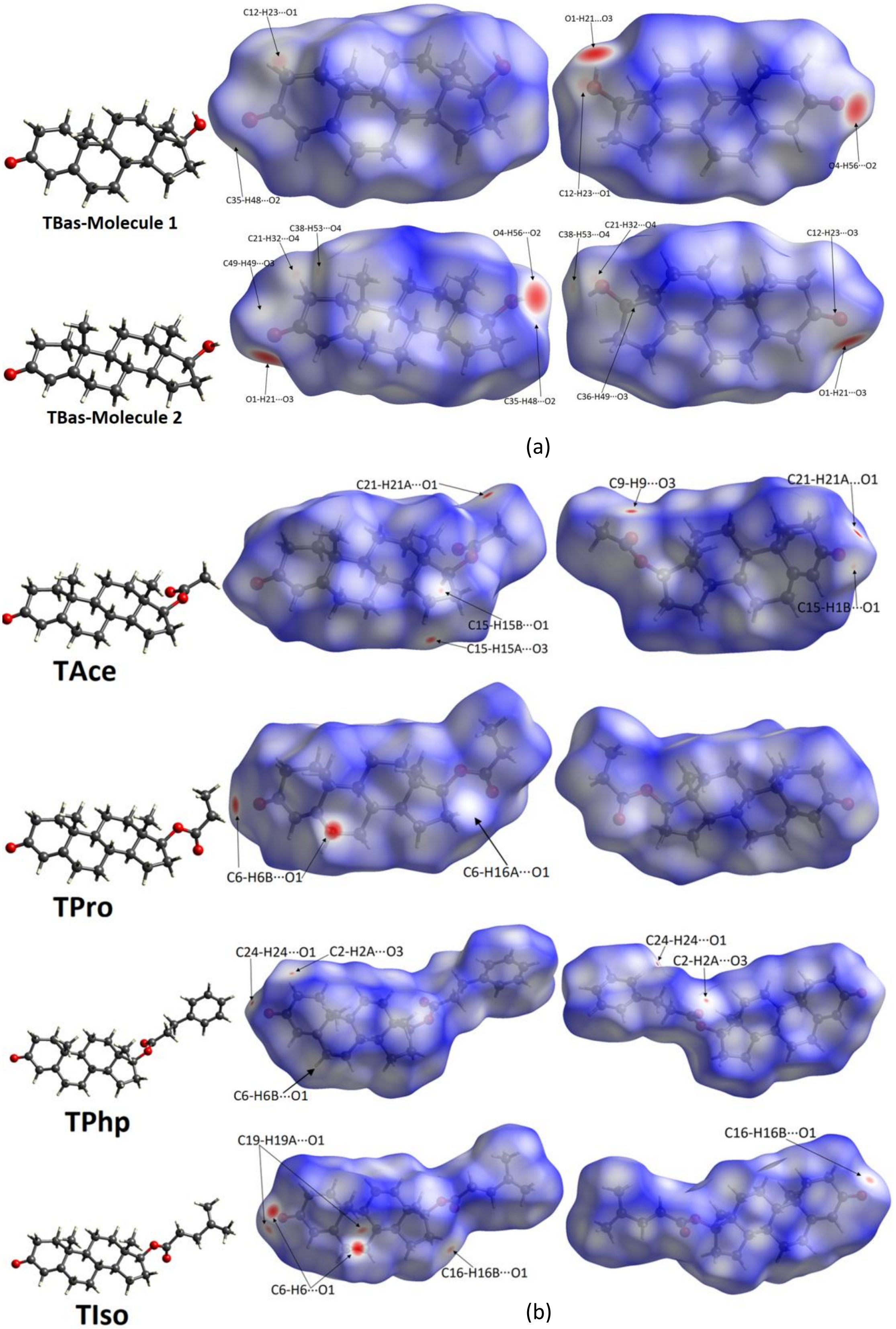

Figure S3. Views of the Hirshfeld surfaces mapped with d_{norm} ilustrating the intermolecular contacts referred in Table S1: TBas (a) and studied esters (b). Surfaces are represented with the clour scale in the ranges as follows: -0.58 (red) to 1.61 (blue) for TBas, -0.08 (red) to 1.36 (blue) for TAce, -0.18 (red) to 1.55 (blue) for TPro, -0.03 (red) to 1.51 (blue) for TPhp. -0.2 (red) to 1.57 (blue) for TIso

Table S1. *Hydrogen bond geometry for analyzed crystals (Å, °)*

**Structure**	**D-H···A**	**D-H**	**H···A**	**D···A**	**<(D-H···A)**
TBas	O1-H21…O3	0.993	1.849(5)	2.816(3)	163.7(3)
	C12-H23…O1	1.089	2.461(1)	3.528(5)	166.3(2)
	C12-H23…O3	1.089	2.664(4)	3.326(1)	118.6(5)
	C20-H29…O2	1.089	2.661(2)	3.391(1)	123.8(4)
	O4-H56…O2	0.993	2.005(4)	2.982(3)	167.4(5)
	C35-H48…O2	1.089	2.615(2)	3.353(2)	141.4(1)
	C21-H32…O4	1.089	2.525(3)	3.481(1)	145.8(7)
	C38-H53…O4	1.089	2.512(2)	3.601(1)	178.1(3)
	C36-H49…O3	1.089	2.548(2)	3.506(4)	142.0(1)
TAce	C21-H21A…O1	1.089	2.486(5)	3.452(3)	147.1(3)
	C15-H15B…O1	1.089	2.563(1)	3.588(1)	156.5(4)
	C15-H15A…O3	1.089	2.502(2)	3.313(4)	130.4(2)
	C9-H9…O3	1.089	2.716(4)	3.627(2)	140.9(4)
TPro	C6-H6B…O1	1.089	2.355(2)	3.373(5)	154.9(1)
	C16-H16A…O1	1.089	2.618(3)	3.686(4)	166.6(5)
TIso	C6-H6B…O1	1.089	2.336(3)	3.394(3)	162.6(4)
	C16-H16B…O1	1.089	2.485(5)	3.414(8)	142.6(7)
	C19-H19A…O1	1.089	2.456(2)	3.491(2)	157.5(2)
TPhp	C24-H24…O1	1.089	2.547(5)	3.472(1)	142.2(8)
	C2-H2A…O3	1.089	2.714(2)	3.201(3)	106.6(9)
	C6-H6B…O1	1.089	2.667(6)	3.367(5)	121.5(1)

Table S2 . *Solubility of analyzed testosterone esters in various oil solutions*

	**APRICOT (mg/mL)**	**GSO (mg/mL)**	**SESAME (mg/mL)**	**MCT (mg/mL)**	**CASTOR (mg/mL)**	**COTTON (mg/mL)**	**Half-life (days)**
TAce	82.5	98.8	99.0	120.5	162.1	98.8	less than one day
TPro	127.3	152.2	188.7	185.4	194.3	174.0	1 day

TPhp	116.5	143.4	160.8	197.5	209.6	155.8	2.5 days
Tiso	370.0	387.1	460.0	438.7	489.3	479.8	3.1 day
